# Asymmetry of cerebral gray and white matter and structural volumes in relation to sex hormones and chromosomes

**DOI:** 10.3389/fnins.2014.00329

**Published:** 2014-11-27

**Authors:** Ivanka Savic

**Affiliations:** Stockholm Brain Institute, Department of Women's and Children's Health and Neurology Clinic, Karolinska Institute and Karolinska HospitalStockholm, Sweden

**Keywords:** cerebral asymmetry, X-chromosome, sex hormone, gender, MRI

## Abstract

Whilst many studies show sex differences in cerebral asymmetry, their mechanisms are still unknown. This report describes the potential impact of sex hormones and sex chromosomes by comparing MR data from 39 male and 47 female controls and 33 men with an extra X-chromosome (47,XXY).

**Methods:** Regional asymmetry in gray and white matter volumes (GMV and WMV) was calculated using voxel based moprhometry (SPM5), by contrasting the unflipped and flipped individual GMV and WMV images. In addition, structural volumes were calculated for the thalamus, caudate, putamen, amygdala, and hippocampus, using the FreeSurfer software. Effects of plasma testosterone and estrogen on the GMV and WMV, as well on the right/left ratios of the subcortical volumes were tested by multi-regression analysis.

**Results:** All three groups showed a leftward asymmetry in the motor cortex and the planum temporale, and a rightward asymmetry of the middle occipital cortex. Both asymmetries were more pronounced in 46,XY males than 46,XX females and 47,XXY males, and were positively correlated with testosterone levels. There was also a rightward asymmetry of the vermis and leftward GMV asymmetry in the cerebellar hemispheres in all groups. Notably, cerebellar asymmetries were larger in 46,XX females and 47,XXY males, but were not related to sex hormone levels. No asymmetry differences between 46,XX females and 47,XXY males, and no overall effects of brain size were detected.

**Conclusion:** The asymmetry in the planum temporale area and the occipital cortex seem related to processes associated with testosterone, whereas the observed cerebellar asymmetries suggest a link with X-chromosome escapee genes. Sex differences in cerebral asymmetry are moderated by sex hormones and X-chromosome genes, in a regionally differentiated manner.

## Introduction

Laterality of the human cerebral hemispheres has profound implications for higher cognitive functions and behavior making both functional and structural asymmetries of the brain utterly intriguing (Toga and Thompson, 2003).

Numerous studies have revealed the consistent presence of functional asymmetries and some of these seem to correspond with structural asymmetries (Toga and Thompson, 2003; Narr et al., 2007). Among the structural asymmetries generally found in adults are a larger right hemisphere than left, which mainly seems to be attributed to white matter volume (WMV), and fronto-occipital asymmetry (torque), where the right prefrontal cortex gray matter volume (GMV) is greater than the left, and the left occipital GMV is greater than the right (Good et al., 2001; Raz et al., 2001, 2004; Hamilton et al., 2007; Rezaie et al., 2009). Computed tomography and magnetic resonance imaging (MRI) studies have shown that these asymmetries are more prominent in right-handers (Bullmore et al., 1995; Hamilton et al., 2007). Cerebral tissue asymmetry has been detected at birth and even as early as at 22 weeks of gestational age (Chi et al., 1977; Hering-Hanit et al., 2001), and is, thus, an early phenomenon in the development of the human brain.

Regional structural and functional asymmetries were initially primarily described for language networks with the striking structural asymmetry of the inferior frontal gyrus (Broca's area) and the planum temporale (Wernicke's area) (Geschwind and Levitsky, 1968; Galaburda et al., 1990). Subsequently, with the application of MRI, several other regional asymmetries have been recognized. For example, left > right asymmetry has been reported in the precentral gyrus, and right > left asymmetry in the cingulate sulcus, the uncus, the anterior insular cortex, the superior temporal sulcus, the caudate nucleus, and the dorsal thalamus (Good et al., 2001; Watkins et al., 2001; Luders et al., 2004; Herve et al., 2005, 2006, 2009). In addition, left > right asymmetry has been found in the putamen, and right > left asymmetry in the hippocampus (Raz et al., 2001, 2004); see also Hou et al. for a comprehensive review (Hou et al., 2013). Some of the aforementioned asymmetries (hippocampus, planum temporale) exist in non-human primates as well (Lyn et al., 2011); they also seem to be independent of the intracranial volume (Barrick et al., 2005) and are believed to reflect adaptation to a challenging environment. Despite rather extensive research, little is known about their underlying mechanisms. Unraveling these mechanisms is both clinically and theoretically important, as many higher cognitive functions are organized along the left/right axis, and a number of developmental disorders (e.g., dyslexia, autism, schizophrenia, ADHD) are associated with reductions of the normal structural asymmetry (Guerguerian and Lewine, 1998; Schulte-Korne et al., 1999; Irle et al., 2005; Shaw et al., 2009). Recent data from subjects with posttraumatic stress disorder (PTSD) suggest that this reduction may also occur in response to stress (Irle et al., 2005; Kim et al., 2012). Changes in cerebral symmetry may, thus, lead to abnormal functional organization and impaired functioning (Toga and Thompson, 2003).

In the quest to discover the biological underpinnings of cerebral asymmetry, important information may potentially be extracted from studies of sex differences. Right > left hemispheric asymmetry is, according to several reports, more pronounced in adult males than females (Nopoulos et al., 2000; Savic and Lindstrom, 2008). A right > left asymmetry has more consistently been reported in the hippocampus volume (Giedd et al., 1996; Pfluger et al., 1999; Toga and Thompson, 2003; Gilmore et al., 2007; Savic and Arver, 2011) and seems detectable as early as the human fetus stage (de Lacoste et al., 1991)—although there are also studies that failed to detect such differences (Hering-Hanit et al., 2001). There are also reports of sex differences in structural asymmetry of the cerebellum (Fan et al., 2010; Tiemeier et al., 2010).

Sexual dimorphism exhibited in brain asymmetry may be related to the effects of sex hormones, sex chromosome genes, environmental factors, or a combination of the three. Based on animal studies, it has long been believed that cerebral sex differences are linked to prenatal androgen levels and the post-pubertal androgen surges (Giedd et al., 2006; van Rijn et al., 2008; Rezaie et al., 2009; Steinman et al., 2009). The androgen theory, as the sole explanation to structural asymmetries of the brain, has been challenged by findings of asymmetrical expression in some genes of the human embryonic cortex after only 12 weeks of gestation (Sun et al., 2005), that is, before the onset of gonadal production of sex hormones. That genetic programs may also be operating in the development of asymmetry is suggested by studies of patients with sex chromosome aneuploidy, showing left hemisphere atrophy in language processing regions in men with Klienefelter's syndrome (47, X46 XY men) and right parietal lobe atrophy in visuospatial processing regions in women with Turner's syndrome (X0 women) (Baron-Cohen, 2004; Lentini et al., 2013; Savic and Arver, 2013). Subjects with X-chromosome aneuploidy have also shown changes in functional lateralization during language tasks, and a reduced activation of the left superior temporal gyrus and the supramarginal gyrus has been observed in 47,XXY men (van Rijn et al., 2008). A study of 47,XXY men also showed larger right > left asymmetries with regard to non-verbal right-hemispheric tasks (Netley and Rovet, 1984). Furthermore, a more recent study including subjects with Turner's as well as Klienefelter's syndrome suggests that the number of sex chromosomes influences the development of brain asymmetry in a differentiated manner along the antero-posterior axis (Rezaie et al., 2009).

The exact patterns of asymmetry in the gray and white matter of separate brain structures along the antero-posterior axis, however, have not previously been investigated exploratively, and it is possible that both structural and functional asymmetries of some structures could be attributable to sex chromosome-linked processes, whereas others could be affected by sex hormone levels. The present study was designed to address this issue by comparing MRI data from 46,XX females, 46,XY males, and men with Klinefelter's syndrome, 47,XXY. In a recent study, this type of experimental design was shown to be suitable for investigations of the possible effects of sex hormones and sex chromosomes on cerebral tissue (Lentini et al., 2013; Savic and Arver, 2013). Based on our previous reports, which showed several structural asymmetries among healthy controls (Savic and Lindstrom, 2008; Savic and Arver, 2011), special interest has been paid in the present study to the asymmetry of the structural volumes of the caudate, putamen, thalamus, amygdala and hippocampus, and the possible asymmetry of the entire hemispheric volumes. Both voxel-brain morphometry and structural volumetry have been carried out, and blood samples were drawn for analyses of bioactive testosterone and estradiol. The study also includes measurements of the length of the second and fourth digit, known as the 2D:4D ratio. According to several scientific reports, this ratio, especially on the right hand, may serve as a proxy for fetal testosterone (Manning et al., 1998, 2004; Williams et al., 2000; Lutchmaya et al., 2004; Coates et al., 2009; Honekopp and Watson, 2010). A more detailed reasoning for use of the 2D:4D ratio is described in some of our previous studies (Lentini et al., 2013; Savic and Arver, 2013).

Men with Klinefelter's syndrome are born with one or more extra X-chromosomes. Their phenotype is characterized by hypogonadism. Testosterone levels in men with Klinefelter's syndrome are usually normal or subnormal during the prenatal period, but become significantly reduced in relation to control boys during puberty (Carson et al., 1982; Ratcliffe et al., 1994; Niznikiewicz et al., 2000; Aydin et al., 2005; Aksglaede et al., 2006). 47,XXY males are heterosexual, have male identity, and their gender roles (van Rijn et al., 2008).

It is well known that in the somatic cells of 46,XX females, one of the two X-chromosomes is randomly inactivated. However, about 15% of X-linked genes (so called escapee genes) escape this process and will be expressed from both X-chromosomes. Because only a few of the escapee genes have homologs on the Y-chromosome (Ashburner and Friston, 1999; Xu et al., 2002), a major portion of these X-linked genes will be expressed in excess in 46,XX females. They could, thus, play an important role in sexual differentiation.

Based on this information, it is reasonable to expect differences in regional asymmetries between 47,XXY males and male controls. As discussed in our earlier publications (Lentini et al., 2013) these differences could be primarily related to two types of genetic mechanisms.

- In 47,XXY males there could be an excessive expression of genes that lie in the pseudoautosomal regions of the X-chromosome. Those X-escapee genes that have active Y-chromosome homologs will be expressed in a higher dose in 46,XXY males than in 46,XX and 46,XY controls, potentially leading to differences in cortical thickness in *relation to both control groups*.
- Second, because 47,XXY males may also have X-escapee genes that do not have Y-chromosome analogues (Vawter et al., 2007), these genes will be expressed in excess in 47,XXY males only in relation to 46,XY males but *not in relation to 46,XX females*.

Regional asymmetry was first compared between male and female controls. Next, correlation analyses were carried out amongst the controls with respect to asymmetry ratios (right vs. left) and the z-transformed estrogen and testosterone levels, as described previously (Lentini et al., 2013; Savic and Arver, 2013). Finally, it was investigated whether the regional asymmetry in 47,XXY males differed from those of 46,XY males and 46,XX females, and whether 47,XXY males exhibited a same sex, opposite sex, or entirely singular pattern of asymmetry (different from both control groups).

One hypothesis was that 46,XY males would have more pronounced asymmetries than 46,XX females and that this difference, at least in some parts of the brain, could be related to testosterone levels. A further hypothesis was that 47,XXY males would have less pronounced asymmetries than 46,XY males and perhaps also 46,XX females. The underlying rationale was based on the previous observation that 47,X XY males have reduced left lateralization in dichotic listening tasks (Kompus et al., 2011) and on the finding of reduced leftward lateralization (measured with fMRI) during a language task in this population (van Rijn et al., 2008).

It was further hypothesized that asymmetries that were not linked to sex hormone levels among the controls, and that were also similar to those of 46,XX females and 47,XXY males but different from those of 46,XY males, could be related to genes located on the extra X-chromosome, the so-called escapee genes, which do not have Y-chromosome homologs. Furthermore, those asymmetries that were different in 46,XXY males in comparison with both control groups would indicate processes related to the X-escapee genes, which have Y-chromosome homologs; they would, thus, be associated with trisomy (three sex chromosomes), or represent downstream effects of this type of sex-chromosome aneuploidy.

## Materials and methods

### Population

The population consisted of 33 47,XXY males (age 39 ± 11 years, range 21–50, education 13 ± 3 years), 39 46,XY males (age 35 ± 7 years, range 25–50, education 16 ± 2 years), and 47 46,XX females (age 35 ± 7 years, range 22–50, education 16 ± 2 years), and has also partly been described in our previous studies (Lentini et al., 2013; Savic and Arver, 2013). Due to poor MRI image quality in two subjects the analysis included imaging data from 31 47,XXY males. The 47,XXY males were recruited from the Center of Andrology, Department of Medicine, Karolinska University Hospital, Sweden, the controls from the general public. The age range was 20–50 years, and all participants were right-handed (Edinburgh Handedness Inventory, Oldfield, 1971; Kinsey et al., 2003), and heterosexual (scored Kinsey 0) according to Kinsey's Heterosexual/Homosexual Scale (Kinsey et al., 2003).

Exclusion criteria included having karyotypic mosaicism (assessing the metaphase chromosomes in cells derived from whole blood according to standard procedure), and having a heredity for, history of, or current psychosis. Furthermore, we excluded subjects with a neurological disease, ADHD, personality disorder, major or bipolar depression, alcohol or substance abuse problems. Mild dyslexia was present in about 60% of the 47, XXY men, but no controls.

Two of the 47,XXY males were diagnosed as adults during the course of infertility investigation, the others were diagnosed in early adolescence. All but two (who were deemed to not need testosterone) had been receiving testosterone supplementation subsequent to the diagnosis and were in treatment at the time of the study. The starting age of testosterone treatment ranged from 15 to 40 years. The Ethics Committee of the Karolinska Institutet Stockholm approved the study, and prior to study all participants signed informed consent forms.

### Finger ratios

The 2D:4D ratios of both hands were measured using a steel vernier caliper. Measurements were carried out directly on the fingers, on the ventral side of the hand between the basal crease and the fingertip (Manning et al., 1998, 2004; Lutchmaya et al., 2004; Manno, 2008; Ciumas et al., 2009). As in our earlier study (Savic and Arver, 2013) the 2D:4D ratios of 15 subjects were independently measured by two raters, and the inter-rater correlation was calculated with linear regression (Pearson's coefficient, *p* < 0.05).

### Venous blood samples

Venous blood samples were collected from all the subjects between 8 and 10 a.m. in the morning. Plasma testosterone levels, (nmol/L), 17β-estradiol (pmol/L) (radioimmunoassay, Testosterone RIA DSL-4000, Diagnostic Systems Laboratory Inc., TX), and the sex hormone binding globulin (SHBG) were analyzed in the Chemical Diagnostics Laboratory at the Karolinska University Hospital. The levels of bioavailable testosterone (nmol/L) were calculated using an equation developed by Sodergard et al. (1982). Before conducting the statistical analyses of possible correlations between hormone levels and regional asymmetry, the individual levels of 17β-estradiol and bioactive testosterone were z-transformed within each sex group, because natural sex differences in plasma testosterone levels and 17β-estradiol (pmol/L) could lead to false correlations—considering that the respective hormone values would be located at the two extremes of the correlation slope.

### MRI

#### MRI data acquisition

All the subjects were investigated in a whole-body 1.5-Tesla MRI medical scanner (General Electric, Milwaukee, Wisconsin) equipped with 8-channel phased array receiving. The MRI protocol included the following scans: (1) 3D-weighted T1 SPGR images with 1 mm isotropic voxel size according to a previously described protocol (Ciumas et al., 2010); (2) 2D T2-weighted fast spin echo (FSE) images in the axial plane (effective *TE* = 56 ms, *TR* = 2500 ms, FOV = 24 cm, 23 slices of 3 mm thickness). The latter images were not used in the present report.

#### Voxel-based morphometry

Voxel-based morphometry (Ashburner, 2007) was performed using the Gaser Toolbox (http://dbm.neuro.uni-jena.de/vbm/) with SPM 5 (The Wellcome Department of Imaging Neuroscience, University College London; www.fil.ion.ucl.ac.uk/spm/) and Matlab 7.3 (Math Works, Natick, MA), as described in several related studies, and using The Diffeomorphic Anatomical Registration Through Lie Algebra toolbox (DARTEL, Wellcome Department of Imaging Neuroscience, University College London, UK; http://www.fil.ion.ucl.ac.uk/spm). All the segmented were modulated (multiplied) by the Jacobian determinants, which allowed direct comparisons of regional differences in the volume of each tissue type.

All the T1-weighted MR images were segmented into gray matter (GM) and white matter (WM), and CSF. The segmented images were then flipped vertically in the midsagittal plane (*x* = 0). Next, both the original and the flipped images were imported to DARTEL to create templates (GM and WM). The GM and WM templates of the entire study group were then normalized to MNI space using the 12-parameter transformation and then flipped vertically in the midsagittal plane (*x* = 0), and a mean image of the original and of the flipped GM templates was created (a mean symmetrical template). The individual images were finally normalized to the symmetrical template and finally smoothed with a 8 mm FWHM kernel.

Within-group asymmetries in regional GMV and WMV were tested through paired *t*-tests for each group (46,XX, 46,XY and 47,XXY), comparing the individual, MNI normalized, and segmented GM and WM unflipped images with the corresponding flipped images. Significant clusters were calculated for each subject group with peak threshold at *p* = 0.001, FDR corrected at *p* < 0.01 (the more restrictive significance level was used to avoid large confluent clusters).

Between-group asymmetries were tested with a full-factorial design using each group's (46,XX, 46,XY and 47,XXY) unflipped and flipped normalized images as the factor of variance, and using age and total tissue volume (TV) as the covariates of no interest (peak threshold at *p* = 0.001, FDR corrected at *p* < 0.05). TV was calculated as the sum of individual WMV and GMV. The following contrasts were investigated:

$$\begin{array}{l} \left(46,{\text{XX}}_{\text{unflipped}}-\text{46},{\text{XX}}_{\text{flipped}}\right)\\ \;-(\text{46},{\text{XY}}_{\text{unflipped}}-\text{46},{\text{XY}}_{\text{flipped}})\\ \left(46,{\text{XX}}_{\text{unflipped}}-\text{46},{\text{XX}}_{\text{flipped}}\right)\\ \;-(\text{47},{\text{XXY}}_{\text{unflipped}}-\text{47},{\text{XXY}}_{\text{flipped}})\\ \left(46,{\text{XX}}_{\text{unflipped}}-\text{46},{\text{XX}}_{\text{flipped}}\right)\\ \;-(\text{46},{\text{XY}}_{\text{unflipped}}-\text{46},{\text{XY}}_{\text{flipped}}) \end{array}$$

Each contrast was also run in the opposite direction.

To investigate possible commonalities between two groups vs. the third, conjunctional analyses was used. The following conjunctions were investigated: (46,XX_unflipped-flipped_ − 46,XY_unflipped-flipped_) and (47,XXY_unflipped-flipped_ − 46,XY_unflipped-flipped_); (46,XY_unflipped-flipped_ − 46,XX_unflipped-flipped_) and (47,XXY_unflipped-flipped_ − 46, XX_unflipped-flipped_); (46,XX_unflipped-flipped_ − 47,XXY_unflipped-flipped_) and (46,XY_unflipped-flipped_ − 47,XXY_unflipped-flipped_). Based on previous observations from a similar population, it was assumed that the pattern and degree of asymmetry would be similar among 46,XX females and 47,XXY males and different from that 46,XY males. Therefore, the threshold level of *p* < 0.05 uncorrected was used when testing conjunction (46,XX_unflipped-flipped_ − 46,XY_unflipped-flipped_) and (47,XXY_ů*nflipped-flipped*_ − 46,XY_unflipped-flipped_), and *p* < 0.05 corrected was used for the remaining two conjunctional tests.

Possible asymmetry correlations with bioactive z-testosterone, z-estradiol, and right hand 2D:4D ratios were tested with multi-regression analyses using both control populations, employing age and individual tissue volumes as the covariates of no interest.

***Evaluation of structural volumes***. Segmentation generated with FreeSurfer was used to derive volumes of the total intracranial volume and the volumes of five subcortical structures: the amygdala, hippocampus, caudate, putamen, and thalamus [FreeSurfer software (www.surfer.nmr.mgh.harvard.edu), according to standard procedure, Fischl, 2012]. Data on cortical thickness, which were also generated by the FreeSurfer program, partly from these populations, has been presented in a separate manuscript. The method for generation of subcortical volumes has been described in detail previously (Fischl et al., 2002, 2004; Fischl, 2012; Savic and Arver, 2013). The segmentation masks were first registered to original grayscale images. When necessary, manual correction was carried out by a person educated in neuroanatomy (who had no information about the identity of the subjects).

Possible right/left asymmetries were tested for each separate structure with paired *t*-tests (normally distributed data, *p* < 0.05). Each subcortical volume was divided with the individual intracranial volume, retrieved from the FreeSurfer software. Possible differences in the VOI/total brain volume as well as in the asymmetry index (right/left) of each measured volume between the three groups were tested with One Way ANOVA, after ensuring that the data were normally distributed. Group was used as the between factor (*p* < 0.05). Tukey's statistical test was employed for *post hoc* analyses because the sample sizes were unequal.

***Delineation of the right and left cerebral hemispheres***. The volumes of interest (VOIs) representing the entire right and left cerebral hemispheres were delineated on every second coronal slice of the individual MR images as described previously (Savic and Lindstrom, 2008). Briefly, cerebral hemispheres were divided at the midline in the coronal plane by a hand-drawn line connecting the measured midpoint of the corpus callosum with the midpoint of the hypothalamus, third ventricle, and cerebral aqueduct (Savic and Lindstrom, 2008). Each hemisphere VOI included the ventricles and excluded cerebellum. The right/left hemisphere ratios were tested for normal distribution using the Shapiro–Wilk test. Because they were not normally distributed, group differences in the right/left ratio were tested with the Kruskal–Wallis Test (*p* < 0.05), and in cases of significant group difference, the separate groups were further compared with each other using the Mann–Whitney test (no Bonferroni correction was applied when testing the right/left asymmetry between 46,XX and 46,XY controls, as a significant rightward asymmetry was predicted based on the related studies, Savic and Lindstrom, 2008).

***Correlational analyses***. Possible effects of bioactive testosterone and estrogen and also of right hand 2D:4D ratios on GMV and WMV asymmetry were tested exploratively using VBM, through multivariate linear regression analyses. Z-transformed bioactive testosterone and estradiol and the right hand 2D:4D ratio were used as covariates of interest to examine how the respective correlations with GMV and WMV asymmetry in each voxel differed between groups (one calculation for each factor). Age was employed as the nuisance variable. Based on previous reports about the effects of sex hormones (Bramen et al., 2012; Nguyen et al., 2013; Savic and Arver, 2013), it was hypothesized that correlations would exist between tissue asymmetry and sex hormone levels, and the 2D:4D ratios primarily in regions in which GMV and WMV asymmetry differed between 46,XY males and 46,XX females. The significance level for clusters, which overlapped with those showing differences between 46,XY and 46,XX controls, was therefore *p* < 0.05 uncorrected, whereas a threshold of *p* < 0.05 corrected was employed for the remaining brain regions. Correlation analyses with z-estradiol and z-bioactive testosterone were carried out only among controls, as the corresponding hormone levels in 47,XXY males were biased by testosterone treatment. To the contrary the right hand 2D:4D ratio was used as the covariate in a separate analysis with all three study groups. Possible covariation between asymmetries of the entire hemispheric as well as separate subcortical structural volumes, and z-normalized bioactive testosterone values, estradiol values, as well as the 2D:4D ratios were tested using Pearson partial linear correlation (*p* < 0.05) with age as nuisance variable (SPSS, version 21).

## Results

### Demographic data and digit ratios

Between-subject One-Way ANOVA showed that there was a significant main effect of group on the number of years of education (*F* = 14.1: *p* < 0.001). According to *post-hoc* analysis (Gabriel's procedure), this difference was constituted by the lower education among in the 47,XXY group compared to both male (*p* < 0.001) and female controls (*p* < 0.001), whereas no difference was detected between male and female controls (*p* = 0.91). There were no group differences in regard to handedness or sexual orientation. No gross anatomical abnormalities were found according to an experienced neuroradiologist.

The digit ratio measurements of the two raters were correlated (*r* = 0.93; *p* < 0.001). The results presented here were based on measurements from rater one, as rater two performed ratings for only a portion of the study group. A significant group difference was found for the 2D:4D ratio of the right hand (*p* = 0.015, *F* = 4.5, *df* = 2) but not the left hand (*p* = 0.460, *F* = 0.8, *df* = 2). 47,XXY males and 46,XX females had higher ratios than 46,XY males, while the ratios between 46,XX females and 46,XY males did not differ (Table 1).

**Table 1:** **Demographic data**.

		**46,XX females *N* = 47**		**46,XY males *N* = 39**		**47,XXY males *N* = 33**		
	**Unit**	**Mean**	***SD***	**Mean**	***SD***	**Mean**	***SD***	***F*-value**
Age	Year	35.3	7.4	35.0	6.9	39.0	10.6	2.9
Education	Year	15.9	2.2	15.8	2.4	12.9	2.6	14.1^***^
Right D2:D4^†^	Ratio	1.00	0.03	0.97	0.02	0.99	0.04	4.5^*^
Left D2:D4	Ratio	0.99	0.03	0.98	0.03	0.99	0.04	0.78
Oestradiol (plasma)	pmol/L	150.9	113.0	74.0	28.3	103.3	47.7	7.9^***^
Testosterone (bioactive)	nmol/L	0.5	0.3	6.1	1.5	11.5	6.8	38.3^***^
L hemisphere volume	cm^3^	526.7	56.8	596.8^*^	41.6	565.6	49.2	
R hemisphere volume	cm^3^	525.1	55.2	599.0^*^	41.4	567.6	74.6	

*F-values from group comparisons (One Way ANOVA)*.

* p < 0.05;

*** *p < 0.001*.

*Possible differences in digit ratios between the separate groups were calculated with Tukey post hoc test (p < 0.05)*.

† *Difference between 46,XY and 46,XX, p = 0.042*.

*Difference between 47,XXY and 46,XX, p = 0.998*.

*Difference between 46,XY and 47,XXY, p = 0.041*.

### Within- and between-group asymmetries in regional GMV and WMV

**Figure 1:**
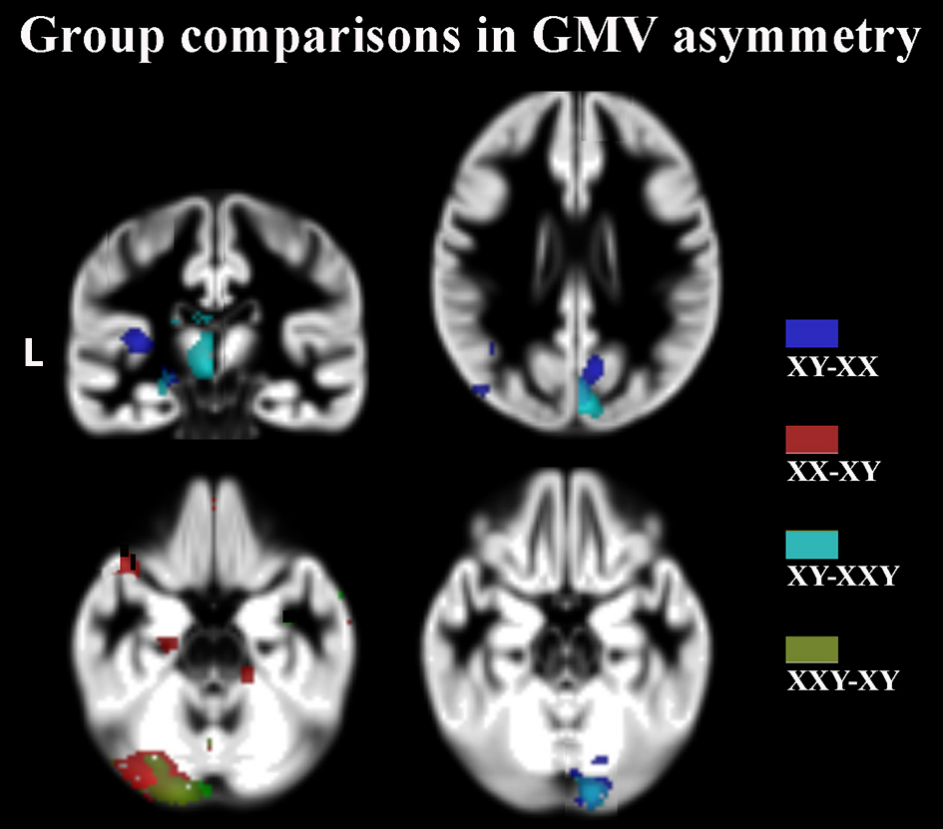
**Clusters indicating significant group differences in the GMV asymmetry, calculated using voxel threshold at *p* = 0.001, corrected at *p* < 0.05, FDR**. Clusters are superimposed on the symmetric GMV template of the entire study group. L, left side.

The data regarding comparisons of regional asymmetries in GMV and WMV are presented in Table 2. In summary, all three groups showed a rightward GMV asymmetry in the anterior cingulate, the superior temporal gyrus, the medial occipital cortex, and the vermis cerebelli. Corresponding asymmetries were found also in WMV, the only exception being that no asymmetry was detected in 47,XXY males in the superior temporal gyrus. There were also leftward asymmetries, which, like the rightward asymmetries, showed a similar distribution amongst all three groups. These asymmetries were detected in the GMV of the motor cortex (covering parts of the planum temporale) (Table 2, Figure 1), in the left cerebellar hemisphere (the semilunar lobules), in the caudate, and in the parahippocampus. The WMV asymmetries largely followed those in the GMV (Table 2).

**Table 2:** **Within group asymmetries in Gray and White matter volume**.

	**Gray matter volumes**			**White matter volumes**		
**Region**	**Z level**	**Size, cm^3^**	**Co-ordinates**	**Z level**	**Size, cm^3^**	**Co-ordinates**
**MALE CONTROLS (RIGHT > LEFT)**						
R superior temporal gyrus	7.8	10.0	46 −28 −2	inf	2.9	52 −18 4
R medial occipital lobe (cuneus)	7.4	7.2	8 −60 10	4.7	0.8	19 −50 0
R anterior cingulate	5.9	10.0	0 26 0	inf	5.8	8 40 −8
	6.2		8 26 −16			0 28 12
R cerebellum (vermis)	7.8	7.8	10 −64 −38	6.8	6.2	28 −58 −26
**MALE CONTROLS (LEFT > RIGHT)**						
L temporo-parietal WM				7.1	2.8	−34 −32 16
L planum temporale + precentral gyrus L caudate	5.7	6.4	−38 −24 8	inf	3.2	−32 24 4
	inf	5.0	−10 6 2			
L lateral occipital lobe (middle occipital gyrus)	6.6	6.2	−32 −96 6	6.7	4.8	−20 −88 −4
L cerebellar hemisphere	inf	28.0	−24 −42 −38	6.0	3.2	−12 −52 −46
**FEMALE CONTROLS (RIGHT > LEFT)**						
R superior temporal gyrus	7.8	7.4	50 −20 −10	4.1	0.2	52 −16 −8
R medial occipital lobe (cuneus)	7.0	4.8	6 −64 14	6.3	3.3	8 −90 14
R anterior cingulate	5.9	13.0	10 60 8	inf	4.8	6 50 −10
R cerebellum (vermis)	6.6	3.2	14 −62 −34	inf	5.2	26 −58 −26
						0 −69 −34
**FEMALE CONTROLS (LEFT > RIGHT)**						
Part of precentral gyrus, planum temporale	5.7	7.7	−40 −28 16	7.4	2.4	−36 26 2
						−32 14 6
L parahippocampus	6.3		−22 −32 −4	4.2	0.4	−14 −34 2
L caudate L inferior frontal WMV	7.5	3.2	−2 10 2	7.4	3.3	−28 22 −12
L middle occipital gyrus				4.8	6.3	−8 −90 14
L cerebellar hemisphere (semilunar lobes)	7.8	12.0	−24 −42 −48			
**XXY (RIGHT > LEFT)**						
R superior temporal gyrus	6.3	6.0	46 −28 −4			
R anterior cingulate	6.3	1.0	10 64 2	7.2	7.2	12 48 −10
R occipital cortex (cuneus)	6.6	7.2	8 −58 10	5.7	13.0	22 −54 18
R Cerebellum (vermis)	6.1	4.0	10 −64 −46	7.5	5.2	18 −48 −26
**XXY (LEFT < RIGHT)**						
L planum temporale + part of the pre and post-central gyrus	6.0	4.9	−42 −22 8	7.5	5.6	−34 −36 10
	5.2	2.0	−26 26 0			−56 −8 26
L caudate	7.2	2.4	−10 10 0			
L parahippocampal gyrus	6.8	9.0	−22 −44 −12			
L inferior frontal gyrus				6.7	2.0	−30 24 −12
L occipital cortex (cuneus)	5.1	1.2	−12 −90 10	5.3	2.8	−8 −90 14
						−14 −88 16
L cerebellar hemisphere, semilunar lobes	6.1	4.0	−40 −56 −20			

*Clusters denoting GM and WM volumes calculated with the SPM5 VBM toolbox, using TV and age as covariates of no interest. Significant clusters calculated using voxel threshold at p = 0.001, FWC corrected at p = 0.01. MNI co-ordinates indicate the peak values and the indicated regions coverage of the respective cluster. R, right; L, left; inf, infinite*.

Interestingly, although the patterns of asymmetry did not differ between the three groups of subjects, there were several significant differences in the degree of asymmetry (Table 3, Figure 1):

- The rightward GMV and WMV asymmetry in the cerebellar vermis and in the medial occipital lobe was significantly greater in 46,XY males compared to both 46,XX females and 47,XXY males. The medial occipital lobe difference could be due to the fact that men generally have greater occipital GMV in the right occipital lobe than women (Lentini et al., 2013).
- In contrast, the leftward asymmetry in GMV and WMV in the cerebellar semilunar lobuli/peducles was more pronounced in 46,XX females and 47,XXY males than in 46,XY males. Group differences in cerebellar asymmetries were not constituted by any particular lateralized reduction in one group vs. the other.
- The leftward asymmetry in the GMV of the planum temporale was more prominent in 46,XY males than in 46,XX females, whereas no significant difference was detected in relation to 47,XXY males.

**Table 3:** **Between group asymmetries in Gray and White matter volume**.

	**Gray matter volumes**			**White matter volumes**		
**Region**	**Z level**	**Size, cm^3^**	**Co-ordinates**	**Z level**	**Size, cm^3^**	**Co-ordinates**
**XY–XX (Right > Left)**						
R middle occipital lobe (lingual gyrus) R cerebellum (vermis)	4.3	0.9	14 −90 −2			
	5.0	1.8	6 −68 −48			
R middle frontal gyrus (and WM)	4.3	1.2	26 66 10	4.3	3.2	13 46 −12
R thalamus				5.2	0.5	2 −4 0
**XY–XX (Left > Right)**						
L planum temporale (and part of inferior motor cortex)	3.7	1.7	−36 −24 10	5.2	0.9	−44 −20 −12
**XX–XY (Right > Left)**						
**XX–XY (Left > Right)**						
L cerebellar hemisphere (GM, WM)	5.4	2.0	−32 −70 −28	5.0	2.1	−32 −54 −22
**XXY–XX (Right > Left)**			–			
**XXY–XX (Left > Right)**			–			
**XXY–XY (Right > Left)**			–			
**XXY–XY (Left > Right)**			–			
L cerebellar hemisphere				3.7	1.7	−38 −68 −32
**XY–XXY (Right > Left)**						
R cerebellar vermis	3.8	0.4	10 −66 −44	4.0	1.8	16 −60 −26
R middle occipital lobe (cuneus)	4.0	0.3	2 −76 17	4.2	0.4	18 −90 20
**XY–XXY (Left > Right)**						
L temporal white matter				4.0	1.1	−42 −20 −14
L parahippocampal GM	4.5	2.4	−24 −40 −38			

*Clusters denoting GM and WM volumes calculated with the SPM5 VBM toolbox, using TV and age as covariates of no interest. Significant clusters calculated using voxel threshold at p = 0.001, corrected at p < 0.05, FDR. Italics denote trend significance (peak value calculated at T = 0.001, corrected p < 0.1, FDR). MNI co-ordinates indicate the peak values and the indicated regions coverage of the respective cluster. R, right; L, left*.

Notably, no differences were found between 46,XX females and 47,XXY males in GMV asymmetry or in WMV asymmetry.

- Finally, conjunctional analyses revealed that the more pronounced leftward cerebellar asymmetry (compared with 46,XY males) was shared between 46,XX females and 47,XXY males. The rightward symmetry in the GMV in the superior temporal gyrus and the GMV of the cerebellar vermis was significantly more pronounced in 46,XY males when compared with both 46,XX females and 47,XXY males. No common clusters were detected with respect to asymmetry of male and female controls in relation to 47,XXY males, nor with respect to both male groups in relation to female controls. Thus, the asymmetry pattern in 47,XXY males was more similar to that of 46,XX females.

#### Relationships between regional asymmetries in the GMV and WMV, sex hormone values, and digit ratios

The explorative multifactorial regression analysis revealed a significant positive correlation between z-transformed levels of bioactive testosterone and the leftward GMV and WMV asymmetry in the planum temporale/motor cortex (*z* = 3.6 cluster sizes 2.0 cm^3^, co-ordinate −42 −8 12 and −40 −42 4 for the GMV, −37 −48 34 for the WMV). Also the rightward asymmetry of the GMV and WMV in the middle occipital lobe was correlated with z-testosterone (*z* = 3.5, cluster sizes 2.0 cm^3^, MNI co-ordinate 20 −70 −16 and 8 −62 22 for the GMV and WMV, respectively).

No significant correlations were detected between the 2D:4D ratios and regional asymmetries in the GMV or WMV.

### Subcortical volumes

The volumes, as well as their right/left asymmetry, were normally distributed (Shapiro–Wilk test). Paired *t*-test showed a significant rightward asymmetry in the thalamus in all three groups (*p* < 0.0001 for 47,XXY, *p* = 0.043 for 46,XY and *p* = 0.006 for 46,XX). The putamen volumes, on the other hand, showed a significant leftward asymmetry in all three groups (*p* < 0.0001 in all the groups). Left caudate volume was larger than the right in 46,XY males (*p* = 0.016), whereas no asymmetry was detected in the two other groups. The right hippocampus was larger than the left in 47,XXY males (*p* = 0.005) and 46,XY males (*p* = 0.013), and no hippocampal asymmetry was found in 46,XX females (*p* = 0.26); finally, no asymmetries could be detected in the amygdala volumes in any of the groups. Subcortical values are presented in Table 4.

**Table 4:** **Subcortical volumes**.

**Region**	**46,XX females**	**46,XY males**	**47,XXY males**
	***N* = 47**	***N* = 39**	***N* = 31**
R Caudate	3.74 ± 0.43	4.21 ± 0.41	3.78 ± 0.43
L Caudate	3.76 ± 0.42	4.27 ± 0.52	3.82 ± 0.43
R Putamen	5.03 ± 0.53	5.56 ± 0.67	4.86 ± 0.49^††^
L Putamen	5.27 ± 0.52	5.88 ± 0.67	5.17 ± 0.52^††^
R Hippocampus	3.95 ± 0.42	4.29 ± 0.36	3.87 ± 0.37^†††^
L Hippocampus	3.93 ± 0.40	4.17 ± 0.45	3.72 ± 0.42^†††^
R Thalamus	6.97 ± 0.67	7.87 ± 0.79	7.17 ± 0.72
L Thalamus	6.85 ± 0.63	7.69 ± 0.68	6.75 ± 0.68^†^
R amygdala	1.47 ± 0.13	1.56 ± 0.17	1.38 ± 0.10^††††^
L amygdala	1.44 ± 0.15	1.55 ± 0.14	1.38 ± 0.13^††††^

*Structural volumes in the respective subject group. Numbers express cm3 (mean and SD)*.

Reductions in 47,XXY subjects in relation to both control groups:

† p = 0.001, F = 12.2,

†† p < 0.001, F = 9.8 (left) and 10.5 (right).

††† p < 0.001, F = 15.6 (left) and 10.5 (right) side.

†††† *p < 0.001; F = 18.6 (left) and 12.1 (right) side*.

*Group difference was detected in the asymmetry of the thalamus (p = 0.001, F = 6.9) and the hippocampus (p = 0.034, F = 3.5). The rightward thalamus asymmetry was more pronounced in 47,XXY males than in both 46,XY males (p = 0.013) and 46,XX females (p = 0.002), without any difference among the two control groups. The right > left asymmetry of the hippocampus was significantly greater in 47,XXY males (p = 0.013) as well as in 46,XY males (p = 0.041) compared to 46,XX females*.

A significant group difference was detected with regard to the asymmetries of the thalamus (*p* = 0.001, *F* = 6.9) and the hippocampus (*p* = 0.034, *F* = 3.5). The rightward thalamus asymmetry was more pronounced in 47,XXY males than in both 46,XY males (*p* = 0.013) and 46,XX females (*p* = 0.002), without any difference among the two control groups. The right > left asymmetry of the hippocampus was significantly greater in 47,XXY males (*p* = 0.013) as well as in 46,XY males (*p* = 0.041) compared to 46,XX females. No asymmetry differences were detected in other structural volumes.

#### Post hoc analysis: group difference with regard to the relative structural volumes (VOI/TBV)

The ratio of each side's structural VOI to total brain volume (TBV) was calculated *post hoc* to investigate whether the observed group differences in asymmetry were attributed to a difference on either particular side. The VOI/TBV ratios were compared between the groups (One-Way ANOVA, *p* < 0.05 with Tukey's *post hoc* test). Group difference was detected for the left thalamus (*p* = 0.001; *F* = 12.2), as a significantly smaller VOI/TBV ratio was found among 47,XXY males compared to both 46,XY and 46,XX groups (*p* < 0.001 for both). Thus, the greater right/left asymmetry in 47,XXY males was probably due to their reduced left thalamus volume. Other group differences were bilateral. They were constituted by lower relative volumes in 46,XXY men in relation to both control groups for the putamen, (*p* < 0.001; *F* = 9.8 and 10.5), hippocampus (*p* < 0.001, *F* = 10.5 and 15.6 for the right and left side) and the amygdala (*p* < 0.001; *F* = 12.1 and 18.6 for the right and left side, respectively), *P* was < 0.001, for all the *post hoc* comparisons.

### Asymmetries in the hemispheric volumes

The paired-sample *t*-test showed a significant difference (asymmetry) between the right and left hemispheric volumes in the male controls [*t*_(39)_ = −2.57; *p* < 0.05), but not in the female controls [*t*_(47)_ = 1.11; *p* = 0.28] nor in the 46,XXY patients [*t*_(31)_ = −1.71; *p* = 0.10], (Table 1). The Kruskal–Wallis test (the asymmetry of hemispheric volumes was not normally distributed) showed that there was a significant group difference in the right vs. left hemispheric volume ratio (Test statistics 7.8, *df* = 2, *p* < 0.019), (Table 1). Subsequent *post hoc* analyses with the Mann–Whitney test showed that this asymmetry was significantly more pronounced in both 47,XXY males (*p* = 0.011) and 46,XY males (*p* = 0.025) than in 46,XX females, whereas no difference was detected between 47,XXY males and 46,XY males (*p* = 0.87).

#### Relationships between digit ratios, sex hormone levels, and hemispheric and subcortical volumes

Because the distribution of the hemispheric data could not be assumed to be normal, Kendall's tau test, a non-parametric correlation analysis, was used. There was a trend, albeit without a statistical significance, toward an inverse correlation between the digit ratio and the right/left hemispheric ratio (*p* = 0.08, all three groups included), indicating that higher exposure to fetal testosterone was associated with more overall rightward hemispheric asymmetry.

None of the calculated subcortical asymmetries were found to be correlated with z-testosterone, z-estrogen, or right hand digit ratio.

## Discussion

The present study was designed to explore possible sex differences in regard to the asymmetry of subcortical structural volumes, and regional gray and white matter volumes. A further purpose was to investigate if and how such asymmetries might be related to sex hormone levels and sex chromosome gene dosage. As in the related studies on the underpinnings of cerebral sex differences, specific comparisons were carried out between 46,XX females, 46 XY males, and 47,XXY males, and included evaluations of possible correlations with sex hormone levels and digit ratios. Asymmetries were detected in all three groups of subjects. They were characterized by greater GMV and WMV in the *right* superior temporal lobe, the cuneus, the anterior cingulate, and cerebellar vermis as well as by a more prominent leftward asymmetry in parts of the precentral gyrus and the planum temporale, and in the left occipital lobe and the cerebellar semilunar lobes (Table 2). Similar findings have been reported in several previous investigations (Falk et al., 1991; Steinmetz, 1996; Good et al., 2001; Watkins et al., 2001; Herve et al., 2006; Takao et al., 2011; Saenger et al., 2012), albeit with some variations. The present results provide additional information to the ongoing discussion on the etiology of cerebral asymmetries by showing that 47,XXY males and 46,XX females have several common features in which they differ from 46,XY men, and by suggesting there may be a regionally differentiated involvement of sex hormonal and sex chromosome related factors. In sum, three types of group differences were found: (1) the leftward GMV and WMV asymmetry of the planum temporale and parts of the motor cortex was significantly greater in 46,XY males than in 46,XX females. The degree of this asymmetry correlated with z-transformed testosterone levels. (2) The rightward asymmetry in the medial occipital GMV and WMV was more prominent in 46,XY males than in 47,XXY males and 46,XX females, without any difference between the two latter groups. Also, the occipital asymmetry was positively correlated with z-testosterone levels. (3) In 46,XY males there was a more pronounced rightward GMV and WMV asymmetry in the cerebellar vermis and a less pronounced leftward GMV and WMV asymmetry in the lateral cerebellum, without any difference between the two latter groups.

The detected leftward asymmetry of the motor cortex and planum temporale is congruent with the well-known lateralization of language functions, and with the previous reports about sex differences in this asymmetry (Geschwind and Levitsky, 1968; Bear et al., 1986; Amunts et al., 2000; Good et al., 2001; Watkins et al., 2001), although they are sometimes small and vary with age (Hirnstein et al., 2013). The observed correlation with z-transformed testosterone levels provides a potential mechanism for the sexually dimorphic character of these regions. Direct correlation between sex hormone levels in men and women and regional GMV and WMV asymmetries has, to the best of our knowledge, not been tested earlier. A recent study by Lombardo et al., however, reports that fetal testosterone levels can predict the GMV of the left planum temporale (Lombardo et al., 2012). Given this, together with the fact that 2D:4D ratios were elevated among the present 47,XXY group, it is somewhat surprising that no correlation was detected between the right hand 2D:4D ratio and the asymmetry of the planum temporale. In general, it is still unclear through which mechanisms the 2D:4D ratio and prenatal androgen exposure are related (Voracek and Loibl, 2009). Contraru to the body of data discussing fetal testosterone exposure, Knickmeyer et al. (2011) suggested that it may be more appropriate to interpret the 2D:4D ratio in adulthood as an *index of early* testosterone exposure rather than prenatal exposure. The failure to find a correlation with digit ratio in the present study should therefore not be taken as an argument against the influence of fetal testosterone on regional cerebral asymmetry.

The detected cerebellar asymmetries, where a more pronounced *anterior* rightward asymmetry was found in males and a more prominent lateral leftward asymmetry in females, deserve a comment. These were not simply based on lateralized group differences; rather, they seem to reflect asymmetry as a distinct factor. Several previous region-of-interest-based volumetric studies have shown right-greater-than-left anterior volume asymmetry and left-greater-than-right posterior asymmetry in a normal cerebellum (e.g., Lawson et al., 2000; Loeber et al., 2001). One study did not find any significant hemispheric asymmetry, possibly because the method relied on manual tracing of only a few sections (Luft et al., 1998). More directly comparable with the present data are the few studies of structural cerebellar asymmetry carried out with VBM. In a detailed VBM study, Fan et al. found that men had an increased rightward asymmetry within lobules I_IV, IX, and Crus I, and decreased leftward asymmetry within lobules VIIb and Crus II (Fan et al., 2010). That the asymmetry of the anterior cerebellum, which connects to the motorcortex, seems to be more pronounced in males accommodates with the more pronounced leftward asymmetry of the motor cortex in males. The lateral cerebellum, on the other hand, connects to the prefrontal cortex and higher cognitive functions, which according to some reports are associated with a sex-differentiated lateralization (Bolla et al., 2004; Meiron and Lavidor, 2013). Considering that no correlation could be detected with sex hormone levels or digit ratios, and that 47,XXY males and 46,XX females differed in a similar manner from 46,XY males, it would be plausible to assume that the observed group differences in cerebellar asymmetry could be linked to X-chromosome gene expression. While such an assumption would need further testing, it should be noted that asymmetries in regional cerebellar volumes begin to show sex differences early in childhood, although the developmental trajectories differ between boys and girls (Tiemeier et al., 2010). A possible X-chromosome dosage effect is also supported by the finding that certain X-chromosome genes are differentially expressed in the male and female cerebellum (Vawter et al., 2004; Abel et al., 2011), and that the number of sex chromosomes influences the development of brain asymmetry in a complex pattern along the antero-posterior axis (Rezaie et al., 2009).

Unlike the cerebellar and cortical asymmetries, those in subcortical volumes showed no correlation with hormone levels or digit ratios, and no pattern of group differences was found to indicate X-chromosome dosage effects. Differences in relation to 47,XXY males detected in the thalamus could be attributed to the left thalamus atrophy in this group that was detected in the present study and the related previous studies (Savic, 2012; Savic and Arver, 2013). The fact that both male groups had a rightward hippocampus asymmetry that was not detected in females and that did not correlate with sex hormone levels or digit ratios adds a new aspect by raising a question about the possible underlying role of the Y-chromosome. Very preliminary data on 46,XY females with complete androgen insensitivity shows a more prominent rightward hippocampus asymmetry compared with female controls, which could be taken as an early indication for testosterone-independent Y-chromosome gene effects. The paucity of relevant literature makes it difficult to make more detailed speculations about the possible underpinnings of the observed group differences in hippocampal asymmetry. A more pronounced hippocampal asymmetry in males has also been found in non-human primates (Murphy et al., 1996; Ragbetli et al., 2002; Hou et al., 2013). An early study of mice reports Y-linked influences on the rightward hippocampal asymmetry (van Abeelen et al., 1989), a finding that needs further investigations.

### Methodological aspects

The volume of gray matter is mainly affected by the number and size of neurons and glia cells. Asymmetric areas have fewer interhemispheric connections than symmetric areas, perhaps as a result of increased axonal pruning (Galaburda et al., 1990).

Among the more specific issues pertaining to the methods used is that the same structure might have a slightly different spatial location in the two hemispheres.

The effect of between hemisphere differences in spatial homology was reduced by the use of a symmetrical template, linear transformations to this template, and by smoothing of the data, as described previously (Good et al., 2001; Luders et al., 2004). Furthermore, the two hemispheres may differ in shape, primarily due to well-known cerebral anticlockwise torques. By employing twelve-parameter transformations (which include skew in addition to the nine parameters of translation, rotation, and scale), the effects of greater extension of the occipital and frontal poles (petalias) in the left and right hemispheres, and, thus, the torques were reduced. According to a study by Narr et al, sex-related differences in hemispheric shape asymmetry are insignificant (Narr et al., 2007). Nevertheless, it cannot be ruled out that occipital torque, which is reported to be more pronounced in males (Barrick et al., 2005) and less prominent in men with Klinefelter's syndrome (Rezaie et al., 2009), could have influenced the observed occipital differences—although more as a reflection of interrelated differences than due to a methodological bias. Of note is also that the occipital asymmetry in the GMV and WMV was found to be correlated with testosterone levels, even within the 46,XY group.

Another important methodological issue pertains to the interpretation of findings from 47,XXY males. Although androgen levels become notably reduced for these individuals during puberty, when the majority of the subjects started testosterone supplementation, it cannot be excluded that the similarities to 46,XX controls could, to a certain extent, be attributed to early androgen deficits.

The 47,XXY population had significantly lower education, which, theoretically, could have affected the results. However, adding education as nuisance covariate did not change the results.

The fact that hormone levels were, as in several previous studies (Neufang et al., 2009; Peper et al., 2009; Witte et al., 2010), measured on only one occasion is a limitation. Hormone levels vary with activity, stress, and sleeping patterns. Special effort was made to standardize these factors, and it can be claimed that the measures of hormone levels and cerebral GMV and WMV were temporally related (blood samples were taken on the same day as the MRI scans). Admittedly, multiple measurements of serum hormone levels over time might, nevertheless have been more precise for determining the link between circulating hormones and brain morphology.

White matter asymmetries largely followed those of the gray matter, which seems biologically plausible. This observation may, however, also reflect an edge effect—if the interface of one tissue compartment were to be displaced, the difference could be appreciated in both compartments. Such effects were not detected by visual inspection of significance maps.

## Conclusion

The present study expands the previous neuroimaging literature on cerebral asymmetries by proposing that processes linked to X-chromosome gene dosage affect the pattern of cerebellar asymmetries, whereas processes primarily linked to testosterone levels seem to influence the asymmetry in the planum temporale and part of the motorcortex as well as in the occipital cortex. By identifying brain areas that seem to exhibit the effects of X-chromosome genes, the present results add to the animal data concerning the genes located on X- and Y-chromosomes that could contribute to sex differences in regional asymmetry. Brain asymmetry is usually regarded to be a developmentally adaptive and aiming at improving the computational efficiency by promoting intrahemispheric processes in larger brains (Hutchinson et al., 2003; Abdul-Kareem et al., 2011; Ellis et al., 2013). By showing similarities between 46,XX women and 47,XXY men, whose brains are not smaller than those of 46,XY controls, the present study shows that other factors than cerebral size moderate cerebral asymmetries. Discussion about functional implications is outside the scope of this paper and would require parallel behavioral and fMRI studies. It is, however, important to emphasize that less pronounced asymmetry in women does not imply impaired performance compared to men, but rather a more bilateral hemispheric activation for comparative functions. This emphasizes the importance of comparing males and females separately, especially when trying to make inferences regarding disease-linked changes in hemispheric asymmetry. Whether reduction of natural asymmetry affects men and women similarly in various neuropsychiatric conditions, and how this affects male and female patients with regard to function is, therefore, an issue needing special attention in future studies.

### Conflict of interest statement

The author declares that the research was conducted in the absence of any commercial or financial relationships that could be construed as a potential conflict of interest.
